# Experimental and Computational Study for the Design of Sulfathiazole Dosage Form with Clay Mineral

**DOI:** 10.3390/pharmaceutics15020575

**Published:** 2023-02-08

**Authors:** Eugenia Moreno-Domínguez, Ana Borrego-Sánchez, Rita Sánchez-Espejo, César Viseras, Claro Ignacio Sainz-Díaz

**Affiliations:** 1 Department of Pharmacy and Pharmaceutical Technology, Faculty of Pharmacy, University of Granada, Campus de Cartuja s/n, 18071 Granada, Granada, Spain; 2 Instituto Andaluz de Ciencias de la Tierra, Consejo Superior de Investigaciones Científicas (CSIC)-Universidad de Granada (UGR), Av. de las Palmeras 4, 18100 Armilla, Granada, Spain

**Keywords:** sulfathiazole, clay minerals, montmorillonite, drug delivery system, solubility, computational calculations

## Abstract

Sulfathiazole is an antimicrobial belonging to the family of sulfonamides, which were the first antibiotics to be discovered. Sulfathiazole is generally administered orally, and its main disadvantage is that it has low aqueous solubility, requiring high doses for its administration. This fact has led to side effects and the generation of bacterial resistance to the drug over time. The improvement of its solubility would mean not having to administer such high doses in its treatment. At the same time, montmorillonite is a natural, low-cost, non-toxic, biocompatible clay with a high adsorption capacity. It is potentially useful as a nanocarrier to design sulfathiazole dosage forms. In this work, the interaction between the drug and the clay mineral has been studied from an experimental and computational atomistic point of view to improve the drug’s biopharmaceutical profile. The results showed the potential enhancement of the drug solubility due to the correct adsorption of the sulfathiazole in the clay interlayer space. As a result of the inclusion of sulfathiazole in the interlayer of the clay mineral, the solubility of the drug increased by 220% concerning the pristine drug. Experimentally, it was not possible to know the number of drug molecules adsorbed in the interlayer space or the external surface of the carrier. Theoretical studies will enable the knowledge of the stoichiometry of the drug/clay hybrids, with three molecules in the interlayer space being the most favorable process. The resultant basal spacing was in agreement with the experimental results.

## 1. Introduction

Montmorillonite (MMT) is a laminar phyllosilicate with a peculiar nanostructure. It has a TOT structure, in which each layer has one octahedral (O) sheet located between two tetrahedral (T) sheets. There are interlayer spaces between the layers, which are capable of holding water and other molecules. This ability of MMT to adsorb molecules, which can also occur by cation exchange [1,2,3], is very relevant in the pharmaceutical field. MMT can be used as an excipient and allows drugs to be adsorbed in the interlayer space of the clay mineral, modifying its properties, such as solubility, release, or stability [4,5,6]. To date, several studies have been conducted to encapsulate drugs in MMT to modify the release profile of the pure drug. The formation of drug-clay complexes in aqueous phase conditions (used to intercalate a hydrophilic drug), as well as in dry conditions or in organic solvents (to adsorb hydrophobic drugs), has been investigated elsewhere [4,7,8]. Another factor in drug release experiments with MMT is the pH of the release medium, as pH modifies the rate of drug delivery. The nature of the drug and the pH of the medium will influence the cation exchange in the interlayer of the clay [7,9,10,11]. In many cases, drug encapsulation has been shown to occur under basic conditions [7].

In particular, studies have also been carried out on poorly soluble drugs to increase the drug dissolution profile after the previous interaction with MMT. For example, the work carried out by Koleman et al. [12] with several combinations of phenytoin-MMT showed that the bioavailability of phenytoin improved in the mixtures with MMT. In addition, this clay mineral showed great potential to enhance the bioavailability of oral glutathione [13]. More recently, Dening et al. [14] demonstrated that MMT-based lipid formulations enhance the bioavailability of blonanserin. Other studies were carried out in which the interaction between praziquantel and MMT clay mineral increased the solubility and dissolution profile of the drug [15,16]. Computational calculations have also been used to study the main interactions between this drug and clay surfaces at the atomistic level, which helps the design and development of new praziquantel delivery systems [17].

**Figure 1:**
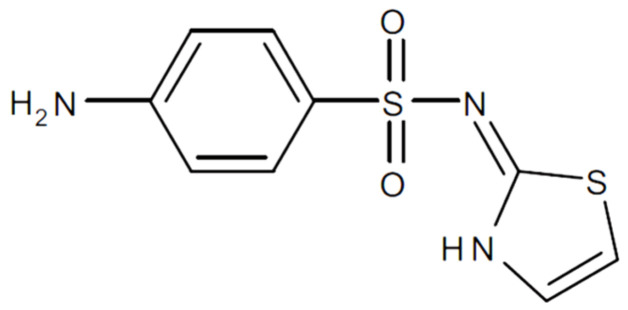
Chemical structure of sulfathiazole.

Therefore, MMT is a good candidate to design a new release system of drugs, such as sulfathiazole (STZ), which is a hydrophobic antimicrobial used to treat oral, ophthalmological, gastrointestinal, and dermatological infections (Figure 1). Specifically, this antibiotic is very effective in treating infections caused by pneumococci, streptococci, and staphylococci. STZ is generally administered orally and presents low aqueous solubility [18,19], so its dissolution is a limiting factor in the adsorption rate [20]. Therefore, it is necessary to administer high oral doses of the drug for effective treatments. In addition, its use in humans has currently decreased due to the appearance of resistance and the discovery and generalization of more effective and less toxic alternatives. Hence, STZ is a target drug for improving its biopharmaceutical profile through novel dosage systems that increase its solubility and reduce side effects and drug resistance.

Over the years, different authors have studied the improvement of the dissolution rate of STZ, an aspect of particular technological interest due to the slightly aqueous solubility of the drug [21,22,23]. Several studies have been carried out concerning the different polymorphic forms of sulfathiazole [24]. The search for a suitable polymorph is essential to ensure the drug’s therapeutic activity. Specifically, five polymorphs of sulfathiazole have been described [25,26]. In addition, studies were carried out at the computational level to analyze the structural and physicochemical properties of the molecule. For example, Density Functional Theory (DFT) calculations were performed to investigate sulfathiazole-theophylline and sulfathiazole-sulfanilamide cocrystals [27].

However, increasing STZ solubility with a clay mineral as an excipient has not been studied to date. The employment of a clay mineral to improve the technological and biopharmaceutical properties of the drug appears to be a novel strategy. Therefore, the aim of this work was to carry out an experimental and computational study to improve the aqueous solubility of STZ using MMT as a low-cost nanocarrier. The results will improve the current treatment with STZ, which requires high doses of the drug. Increasing the water solubility of STZ and consequently reducing the high doses currently used, as well as minimizing the increase in the bacterial resistance to the drug, will be achieved by using a natural and lower cost alternative to the other strategies for increasing solubility.

## 2. Materials and Methods

### 2.1. Materials

Sulfathiazole (STZ) was purchased from the Sigma-Aldrich Company (St. Louis, MO, USA). Pure acetone was used as a solvent. Purified pharmaceutical degree Montmorillonite (MMT), under the trade name of Veegum HS^®^ (MMT), was purchased from Vanderbilt Company (Norwalk, CT, USA).

### 2.2. Experimental Methods

#### 2.2.1. Preparation of Sulfathiazole/Clay Mineral Interaction Products

Following the methodology previously described [15], 0.5 g of STZ was dissolved in 100 mL of acetone. Then, 2.5 g of MMT was dispersed in this solution and kept under magnetic stirring at room temperature for 24 h. The ratio of drug/excipient was 1:5 (*w/w*) to ensure the complete interaction between the components. After 24 h under stirring, the solvent (acetone) was evaporated at 40 °C using a rotary evaporator (Buchi^®^ R II, Flawil, Switzerland), and the solid residue STZ-MMT (also called interaction product or IP) was stored in a laboratory oven at 37 °C.

#### 2.2.2. X-ray Diffraction

X-ray diffraction (XRD) analysis was performed on the MMT samples and oriented IP samples on glass slides (oriented aggregates previously prepared), as well as on the pure drug powder. The measurements were carried out using a Philips^®^ X-Pert model X-ray diffractometer (Marvel Panalytical, Madrid, Spain) with CuKα radiation. The data obtained from the X-ray diffraction were analyzed using the XPOWDER^®^ software (version 2004) [28]. Two replicates of the samples were made.

#### 2.2.3. Thermal Analysis

Thermogravimetric analysis (TGA) and differential scanning calorimetric analysis (DSC) were performed with a Mettler Toledo mod. TGA/DSC1 calorimeter (Mettler Toledo, Barcelona, Spain), equipped with a sensor and FRS5 microbalance (precision 0.1 μg). For TGA runs, the heating rate was 10 °C/min in the 23–950 °C temperature range and, for DSC runs, 5 °C/min in the temperature range of 26–420 °C. Nitrogen was used as purge gas in DSC under 15 mL/min flow.

#### 2.2.4. Scanning Electron Microscopy (SEM)

Microphotographs of the samples were obtained using a Phenom™ G2 pro scanning electron microscope with a voltage of 5 kV and a high-sensitivity backscattered electron detector (Professional Phenom Desk Scanning Electron Microscope, ThermoFisher Scientific, Waltham, MA, USA). The samples were deposited in powder form directly on a previously prepared double-sided carbon adhesive tape on the aluminum button. The images were captured digitally using the program coupled to the microscope (Phenom-World ProSuite Software).

#### 2.2.5. Fourier Transformed Infrared (FTIR) Spectroscopy

The FTIR spectra were recorded in the range 3900–400 cm^−1^ with a 0.5 cm^−1^ resolution and a well-plate sampler by using a JASCO 6200 spectrophotometer (JASCO, Pfungstadt, Germany) with the Spectra Manager II software (version 2).

#### 2.2.6. Elemental Analysis

The samples were analyzed to determine the sulfur and nitrogen components with the Thermo Scientific Model Flash 2000 Elemental Analyzer (Thermo Fisher Scientific, Waltham, MA, USA).

#### 2.2.7. Solubility Studies

The solubility of STZ and STZ-MMT in water was measured by placing 75 mg of the drug (which corresponds in the case of IP to 450 mg of IP) in 10 mL of water (supersaturated conditions). The supersaturated solution was stirred at 26 °C for 72 h in a thermostatic bath. Then, this supersaturated solution was centrifuged, the supernatant was filtered (nitrocellulose membranes, 0.45 μm, Merck Millipore^®^, Darmstadt, Germany), and the drug in the supernatant was quantified by UV spectroscopy at 284 nm (UV–vis spectrophotometer Lambda 25, Perkin Elmer, Waltham, MA, USA). The determined amount of dissolved STZ corresponds to the drug solubility in water. In order to demonstrate the solubility improvement of the STZ in the STZ-MMT IP, the drug solubility of the IP was determined with the same procedure and compared with the STZ solubility. Five replicas of the experiments were performed for the STZ and STZ-MMT samples.

### 2.3. Models

The crystal structure of sulfathiazole (STZ) was taken from The Cambridge Crystallographic Data Center (CCDC) database. Several crystal forms are reported; however, our experimental powder X-ray diffraction corresponds to the crystal form deposited in the CCDC under the access number 1,264,861 [29]. A molecule of STZ was extracted from the crystal to study the molecular structure of the drug. The montmorillonite unit cell was taken from previous works [17], and a montmorillonite supercell was created with 3 × 2 × 1 dimension (MMT). This supercell size was created taking into account the size of the STZ molecule to avoid intermolecular interactions between the drug molecule with the vicinal cells, and periodic boundary conditions were applied. On the other hand, this 3 × 2 × 1 montmorillonite supercell model with formula Na_6_(Al_19_Mg_5_)(Si_47_Al_1_)O_120_(OH)_24_·12H_2_O was created considering the chemical composition of the experimental montmorillonite [15]. Then, different models of adsorption complexes with one, two, three and four molecules of STZ in the interlayer space of the MMT were created to study the most stable structure of each adsorption complex and the main drug-clay interactions. In addition, the obtained experimental interlayer spacing were compared with the theoretical ones to predict how many STZ molecules are experimentally adsorbed in the interlayer space, taking into account the experimental information on the adsorption of the drug in the interlayer of the clay.

### 2.4. Computational Methods

Computational calculations were performed with the Materials Studio software (San Diego, CA, USA) [30]. Calculations based on classical mechanics were conducted using different Force Fields (FF), such as Compass [31], Universal [32], and pcff_interface_v1_5 (INTERFACE) [33], with the Forcite program [30] to optimize the molecular and crystalline structure of sulfathiazole. The atomic charges of the MMT model were set following previous works [34]. The SPC water model was used applying the atomic charges values of 0.41 and −0.82 for H and O atoms, respectively. The atomic charges of STZ were assigned by the FF, considering an electrically neutral molecule.

Additional calculations based on the Density Functional Theory (DFT) of quantum mechanics [35] were also performed to optimize the geometry of the molecular and crystalline structure of the drug. The DMol^3^ code was used with the generalized gradient approximation (GGA) and the revised Perdew-Burke-Ernzerhof functional (RPBE) [36]. In addition, the DFT Semi-core Pseudopotentials (DSPP) [37] and basis set double zeta extended base functions, including the polarization functions (DNP) [38], were used. The convergence threshold in the self-consistent cycle (SCF) of the energy calculation was 10^−6^ Ha and the effect of the dispersion correction of Tkatchenko-Scheffler was included [39].

Subsequently, the crystal structure of MMT was optimized with INTERFACE. The adsorption in the MMT interlayer space of one, two, three and four drug molecules, previously optimized, was studied. The Monte Carlo simulated annealing methods [40] were used for selecting the most stable STZ model in the interlayer space of the MMT as there are many possible conformations of the drug introduced into the clay system. This approach allows a random search of all possible conformations that a drug can adopt and the different dispositions between the drug and clay, thus obtaining the most stable one. The most stable models with one, two, three and four STZ molecules adsorbed on the MMT interlayer were selected and re-optimized at the variable cell with the Forcite program and INTERFACE FF with 3-D periodical boundary conditions.

## 3. Results and Discussion

### 3.1. Solid Characterization

#### 3.1.1. X-ray Diffraction

**Figure 2:**
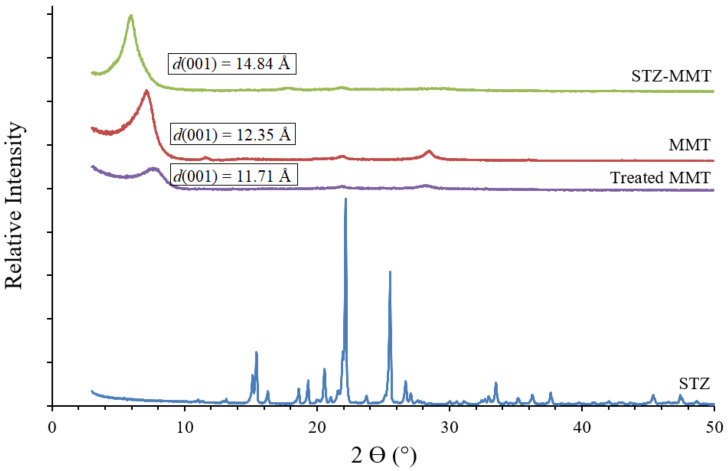
Powder X-ray diffraction patterns of the oriented aggregate of the different samples studied.

The powder XRD patterns of the raw components and the STZ-MMT IP are shown in Figure 2 and a larger view of the graphs is shown in Supplementary Materials (Figure S1). The STZ showed three intense reflections: an intense reflection at around 15.5°, 22°, and 25° 2θ units, corroborating those previously reported [41]. In the diffractogram of the oriented aggregates of the IP, a peak (001) similar to the pristine montmorillonite (MMT) is observed, although shifted to higher *d*-spacing values. This indicates that the interlayer space has increased due to the adsorption of sulfathiazole, corroborating that the drug is present in the IP and its intercalation in the clay. After the interaction of STZ with the MMT, there is a complete absence of drug reflections, which is probably due to a loss of the crystallinity of the STZ. The proportion of the drug in the IP is five times lower than that of the clay and the peaks can be slightly masked, even though STZ is present. In addition, the same IP preparation procedure was performed for the montmorillonite without STZ to corroborate that the increase in the basal spacing of the clay is due to only the drug adsorption and not to the effect of the IP treatment with montmorillonite. In the diffractogram of this sample of montmorillonite, after following the IP preparation procedure without the drug (treated MMT), the interlayer space does not increase, it even seems that there is a tendency to decrease slightly with respect to the untreated MMT, possibly due to a lower water amount (Figure 2).

Therefore, as a result of the interaction between the drug and the clay mineral, the diffraction patterns evidenced significant changes in some of the reflections. The resultant interlayer spaces changed from 12.35 Å in the pristine MMT to 14.84 Å in the STZ-MMT, clearly suggesting the effective intercalation of the drug molecules in the clay mineral interlayer space. Moreover, the treatment without STZ did not produce this spacing shift, *d*(001) = 11.71 Å. This confirms that the increase in the interlayer space in the IP is only due to the STZ molecules being adsorbed in the MMT.

#### 3.1.2. Thermal Analysis

**Figure 3:**
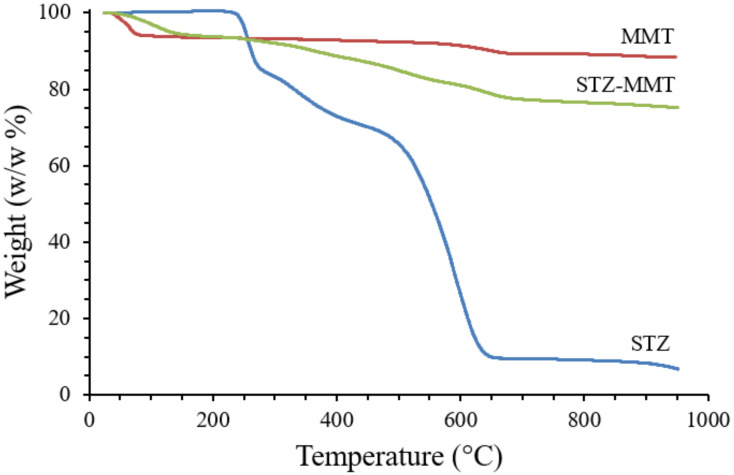
TGA profiles of STZ, MMT and STZ-MMT.

The thermogravimetric analysis profile (Figure 3, and Supplementary Materials Figure S2) shows the weight loss (*w/w*%) of the different samples studied. The drug degrades in a specific temperature range. A weight loss of the STZ is shown in the range 250–950 °C (93.7% *w/w*), where the maximum loss is observed at 640 °C, in accordance with that previously reported [42]. In the MMT, a weight loss of approximately 10% is observed, according to previous studies [15], along with an additional weight loss at 600–700 °C, corresponding to the water formed in the dehydroxylation process of the MMT [43]. The thermal decomposition of the drug is also observed in the weight loss of STZ-MMT in the range 250–950 °C, confirming that the drug is present in the IP and adsorbed on the montmorillonite.

**Figure 4:**
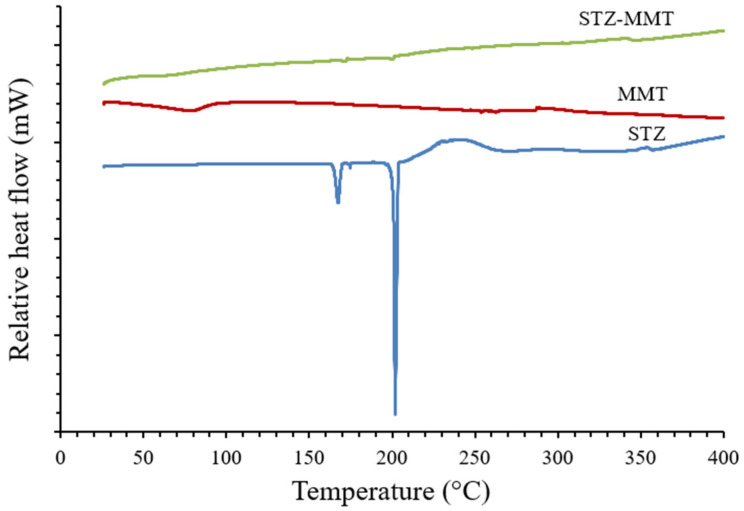
DSC profiles of the studied samples.

The thermal study was completed by also performing a differential scanning calorimetric (DSC) analysis of the samples studied (Figure 4, and Supplementary Materials Figure S3). The DSC profile for the STZ shows a strong endothermic peak at 202 °C, corresponding to the melting point of the drug, which is between approximately 200–203 °C, and two earlier milder peaks at 168 °C and 175 °C, which were also reported previously [44]. At a higher temperature, an exothermic broad peak is observed due to the thermal decomposition of STZ, with weight loss observed above, in the TGA. The MMT showed an endothermic peak around 80 °C, corresponding to the evaporation of the water. As a result of the drug-clay interaction, the DSC curve of the STZ-MMT showed very low intensity peaks of the drug, appearing first as a melting peak at 172 °C, and second at 200 °C. These results show the loss of crystallinity and amorphization of the drug, which may indicate an increase in the solubility of the STZ after its interaction with the clay.

#### 3.1.3. Fourier Transformed Infrared Spectroscopy

**Figure 5:**
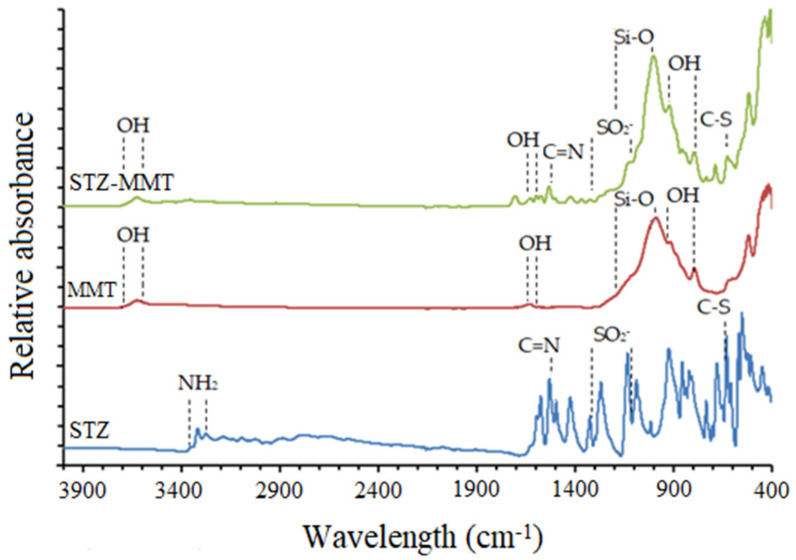
FTIR spectra of STZ, MMT and STZ-MMT, showing the characteristic bands of the samples.

The FTIR spectra of the studied samples, STZ, MMT and STZ-MMT, are compared in Figure 5 (zoomed out in Figure S4). The STZ showed characteristic bands in the range 3320–3279 cm^−1^ assigned to stretching vibration of NH_2_ bonds ν(NH). The bands at 1531 cm^−1^ can be assigned to the ν(C=N) stretching vibration mode. The characteristic bands of the symmetric and asymmetric stretching vibration of the -SO_2_^−^ bond are observed in the 1323–1136 cm^−1^ range. The band corresponding to the frequency vibration of the C-S bond appears at 631 cm^−1^ [45]. Moreover, the spectra of the MMT showed characteristic bands of the clay minerals at 3700–3600 cm^−1^ assigned to the ν(OH) vibration mode of the octahedral OH groups, ν(AlOHAl) ν(AlOHMg), bands at 1680–1600 cm^−1^ of δ(OH) of water molecules, bands at 1200–1000 cm^−1^ of ν(Si-O), and bands at 920–800 cm^−1^ of δ(OH) of the octahedral OH groups [46,47]. In the STZ-MMT, the bands of STZ were detected, which means that the drug is presented in the IP, although no clear differences with the pristine drug were observed due to the high proportion of clay, whose bands overlap those of STZ.

#### 3.1.4. Scanning Electron Microscopy

**Figure 6:**
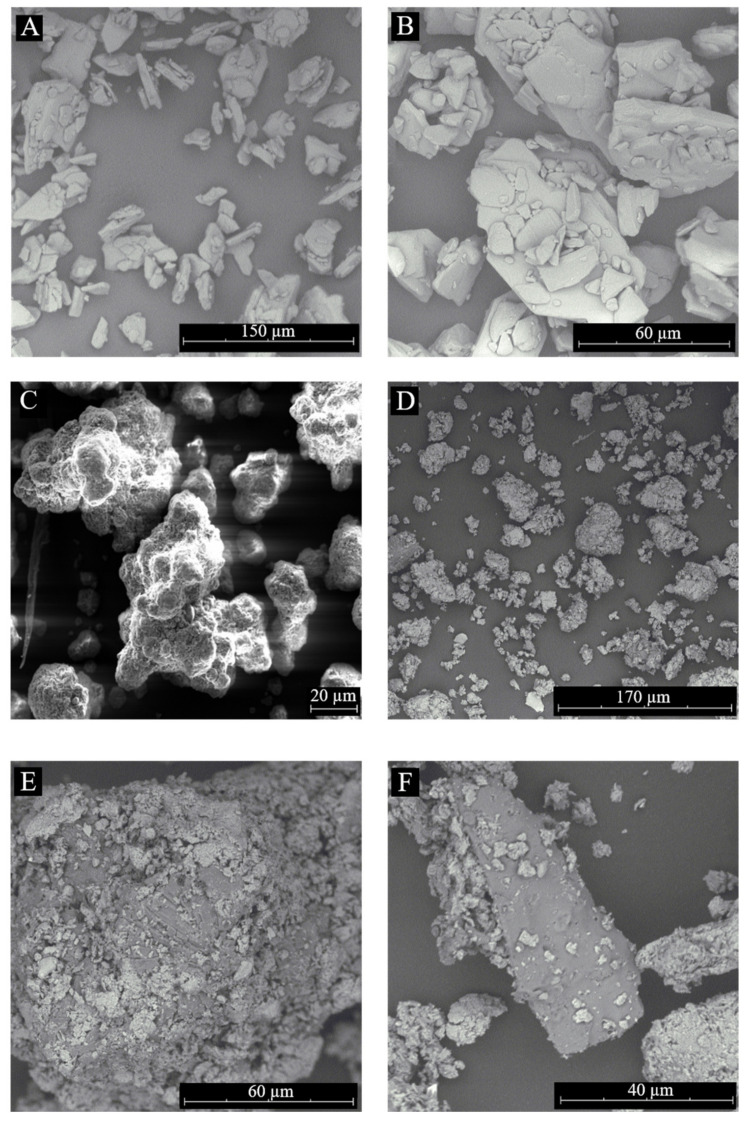
SEM microphotographs of STZ (**A**–**B**), MMT (**C**) and STZ-MMT (**D**–**F**).

The SEM microphotographs are displayed in Figure 6. STZ crystals of different sizes were observed from 5 μm to 90 μm (Figure 6A,B). In Figure 6C, the morphology of the MMT shows grains with different particle sizes and a porous surface. The SEM microphotographs of the STZ-MMT IP (Figure 6D–F) show the porous particles of MMT; however, changes in the texture and morphology of STZ are observed. Figure 6D–F show that there are no pure STZ crystals, and the drug is completely adsorbed on the montmorillonite as molecules or amorphous solid. The loss of the crystalline of STZ is demonstrated as the drug formed aggregates with the montmorillonite particles. These results corroborate the decrease in the drug crystallinity in the IP observed above in the X-ray diffraction and DSC results.

#### 3.1.5. Elemental Analysis

The elemental analysis allowed us to quantify the weight percentage of the nitrogen and sulfur elements present in the drug with respect to the total mass of the sample. The results obtained are observed in Table 1.

The results showed that the weight percentage of nitrogen (N) in the pure drug sample is 17.13%, while in the IP it is 2.66%, as expected, as sulfathiazole was present in one-sixth of the IP because the drug-excipient ratio was 1:5, respectively. In the same way, the weight percentage of the sulfur (S) showed the same results. These results demonstrated that the initial amount of the drug added in the process of preparing the IP is present in the IP and, therefore, no drug was lost during the IP preparation process.

#### 3.1.6. Solubility Studies

In Table 2, the obtained solubility values of the STZ and STZ-MMT are shown. The results showed that the STZ-MMT greatly increases the aqueous solubility of the drug compared to the pristine drug. Specifically, the STZ-MMT IP enhanced the solubility of the drug in 220% compared to the pristine STZ.

### 3.2. Atomistic Computational Analysis

#### 3.2.1. Crystal Structure of Sulfathiazole

**Figure 7:**
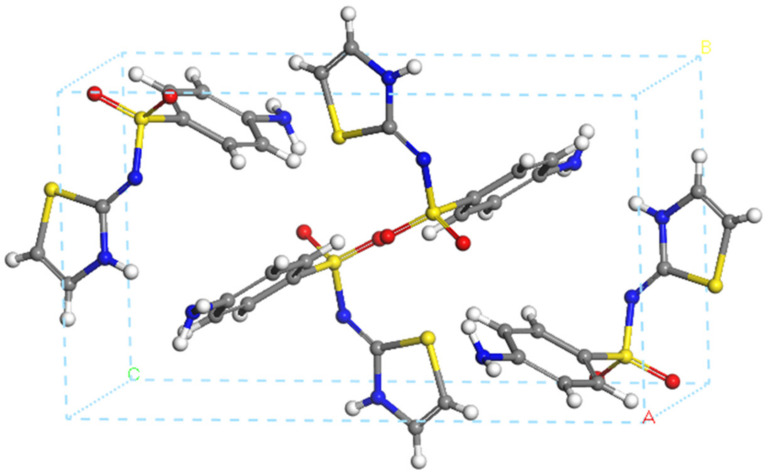
Crystal structure of sulfathiazole. Hydrogen, oxygen, nitrogen, sulfur, and carbon atoms are represented by the colors white, red, blue, yellow, and gray, respectively. A, B, C is X, Y, Z axis, respectively.

The unit cell of the sulfathiazole crystal structure is composed of four sulfathiazole molecules as shown in Figure 7.

The crystal structure of STZ was optimized using different methods. The main bond lengths of the structure are measured and summarized in Table 3. The obtained results indicated that INTERFACE is the one that best reproduces the bond lengths of the experimental sulfathiazole crystal. This result validates the use of this FF for the rest of our work.

#### 3.2.2. Adsorption Modeling in Montmorillonite

The crystal structure of a 3 × 2 × 1 supercell of MMT, Na_6_(Al_19_Mg_5_)(Si_47_Al_1_)O_120_(OH)_24_, was optimized using INTERFACE. The chemical composition of MMT with 12 water molecules and 6 sodium cations per supercell reproduces the experimental sample of MMT [15].

**Figure 8:**
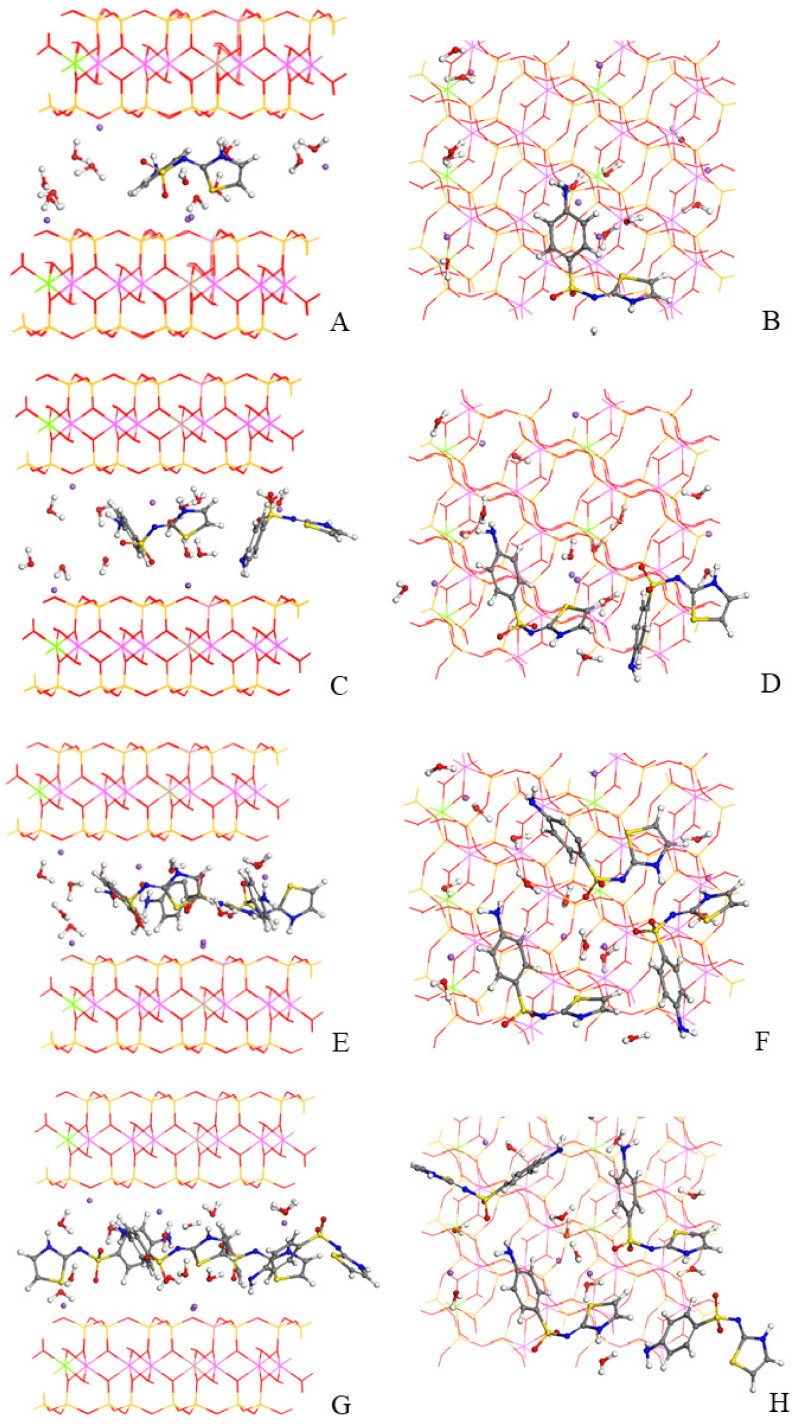
Optimized intercalation models of MMT with one (**A**,**B**), two (**C**,**D**), three (**E**,**F**), and four (**G**,**H**) STZ molecules per supercell, respectively. Views from (010) (**A**,**C**,**E**,**G**) and (001) (**B**,**D**,**F**,**H**) planes. Silicon atoms are represented in yellow, oxygen in red, aluminum in pink, magnesium in green, hydrogen in white, and sodium atoms in bluish-purple.

Experimentally, it was not possible to know the number of STZ molecules that are adsorbed in the interlayer space of the clay and on the external surface of the mineral. The theoretical calculations allow us to compare which adsorption complex corresponds to the experimental basal spacing of the IP. Therefore, the adsorption of one, two, three and four STZ molecules in the MMT interlayer space were studied. To do so, the Monte Carlo simulation method was used with INTERFACE to explore the different conformations and orientations of the drug, water molecules and sodium cations in the interlayer space of the MMT. The most stable model of each complex was selected, which led to us obtaining four adsorption complexes. These complexes were optimized again at variable volumes to calculate the resultant basal spacings (Figure 8).

The *d*(001) spacings of the different sulfathiazole-montmorillonite complexes optimized at variable volumes are compared with the experimental values in Table 4. The amount of three sulfathiazole molecules adsorbed in the interlayer space per 3 × 2 × 1 supercell of the IP has a basal spacing (14.78 Å) and fits best with the experimental one (14.84 Å).

For calculating the adsorption energy of the complexes, we used the formula ΔE_ads_ = E_STZ-MMT_ − (nE_STZ_ + E_MMT_), where the calculated adsorption energy of each of the complexes can be defined as the difference between the energy of the adsorption complex (E_STZ-MMT_) and the energies of the components of the complex optimized separately; that is, the molecule of STZ isolated in a periodical box (E_STZ_) and the montmorillonite supercell (E_MMT_) [17].

The results showed that the adsorption of one or two STZ molecules is an unfavorable process (Table 4). However, in the complexes studied with three and four drug molecules, the adsorption energy is negative, which indicates that, in these cases, the process is energetically favorable (Table 4). Therefore, the theoretical calculations revealed that the adsorption of three drug molecules in the interlayer space of the clay is a favorable process and the basal spacing of the complex is in agreement with the experimental results.

Considering the chemical analysis of the IP samples, we can estimate that the amount of STZ in the final IP solid is 15.5%. Considering the molecular weight of a MMT unit cell with 10% water, we can estimate that there are 3.6 molecules of STZ per supercell. This is slightly higher than the ratio of three STZ in the interlayer space of each 3 × 2 × 1 supercell, as observed above. Therefore, we can conclude that a small proportion of the STZ content in the IP solid remains in the external surfaces of the clay solid, as observed in the SEM micropictures shown in Figure 6.

## 4. Conclusions

In this work, experimental and computational studies have been carried out on the interaction of sulfathiazole with the excipient montmorillonite to obtain a drug-clay system that improves the solubility of the drug.

The experimental study enabled the preparation of the sulfathiazole-montmorillonite interaction product using acetone as a solvent, instead of water, to achieve a higher adsorption, as the drug is poorly soluble in water. The results show that after the interaction with the clay, the drug is adsorbed mainly in the interlayer space of the mineral. Solubility studies have demonstrated that the sulfathiazole-montmorillonite interaction product significantly increases the aqueous solubility of the drug in comparison to the pristine drug.

The study at the atomistic theoretical level has enabled us to obtain the molecular and crystalline structure of sulfathiazole, as well as the different drug-clay adsorption complexes. The analyses showed that the adsorption of three or four drug molecules per supercell in the interlayer space of the clay is a favorable process. Furthermore, modeling studies have indicated, along with the experimental X-ray diffraction results, that the interaction product has three sulfathiazole molecules adsorbed in the interlayer space per supercell.

The correlation of both the experimental and computational studies has allowed a deeper and more detailed insight into the study. This work is the first stage of the design and development of a new system that demonstrates the improvement of the solubility of sulfathiazole after its interaction with clay. This will allow us to design novel pharmaceutical dosage forms in the future, with lower doses of sulfathiazole to reduce toxicity, bacterial resistance and prevent this drug from falling into disuse.

## Figures and Tables

**Table 1:** Elemental analysis of STZ and STZ-MMT IP.

Sample	% N	% S
STZ	17.13	21.61
STZ-MMT	2.66	3.31

**Table 2:** Aqueous solubility values (in mg/mL) of STZ and STZ-MMT IP and increase solubility (in %) with respect to that of STZ (solubility mean values ± 0.01 SD; n = 5).

Sample	Solubility (mg/mL)	Increase (%)
STZ	0.49	
STZ-MMT	1.57	220

**Table 3:** Main values of the bond lengths (Å) of the sulfathiazole crystal after optimization with different computational methods.

Measures	EXP ^a^	UF ^b^	CF ^c^	INTERFACE ^d^	DMol^3 e^
d(H_2_N-C)	1.401	1.425	1.391	1.398	1.371
d(S-C)^1^	1.758	1.812	1.775	1.734	1.744
d(S=O)^1^	1.435	1.545	1.424	1.431	1.482
d(S=O)^2^	1.444	1.546	1.426	1.430	1.486
d(S-N)	1.588	1.780	1.654	1.595	1.638
d(N=C)	1.314	1.295	1.288	1.259	1.321
d(S-CN)^2^	1.741	1.800	1.729	1.772	1.768
d(S-CC)^3^	1.721	1.812	1.731	1.771	1.747
d(N-C)	1.374	1.418	1.367	1.419	1.380

**^a^** EXP: experimental crystal structure [29]; **^b^** UF: calculated with Forcite Universal; **^c^** CF: calculated with Forcite Compass; **^d^** INTERFACE: calculated with Forcite pcff_interface_v1_5; **^e^** DMol^3^: calculated with DMol^3^; d(S=O)_1_: bond close to benzene; d(S=O)_2_: bond close to nitrogen; d(S-C)_1_: bond close to benzene; d(S-CN)_2_: 5-membered ring bond; d(S-CC)_3_ 5-membered ring bond.

**Table 4:** Calculated *d*(001) basal spacings and adsorption energies of the sulfathiazole-montmorillonite complexes.

Complexes	*d*(001) (Å)	Adsorption Energy (Kcal/mol)
(1 STZ)-MMT	13.68	16.74
(2 STZ)-MMT	14.58	1.06
(3 STZ)-MMT	14.78	−38.63
(4 STZ)-MMT	15.65	−58.49
Experimental (Figure 1)	14.84	

## Data Availability

Not applicable.

## Supplementary Materials

The following supporting information can be downloaded at: https://www.mdpi.com/article/10.3390/pharmaceutics15020575/s1, Figure S1: Powder X-ray diffraction patterns of the oriented aggregates of the MMT, STZ-MMT, Treated MMT (**a**) and STZ (**b**) samples; Figure S2: TGA profiles of MMT and STZ-MMT samples; Figure S3: Enlarged DSC profiles of MMT and STZ-MMT; Figure S4: Zoom of the FTIR spectra of STZ, MMT and STZ-MMT.
