# Supplementary material for: Experimental and Computational Study for the Design of Sulfathiazole Dosage Form with Clay Mineral
Source: Pharmaceutics. 2023 Feb 8;15(2):575. doi: 10.3390/pharmaceutics15020575 (PMC9967197; doi:10.3390/pharmaceutics15020575)
Supplement: Supplementary file 1 [file pharmaceutics-15-00575-s001.zip › pharmaceutics-2144098-supplementary.pdf]

pharmaceutics

MDPI

Supplementary Materials: Experimental and Computational Study for the Design of Sulfathiazole Dosage Form with Clay Mineral

Eugenia Moreno-Domínguez [1], Ana Borrego-Sánchez [1,†,*], Rita Sánchez-Espejo [1], César Viseras [1,2] and Claro Ignacio Sainz-Díaz [2]

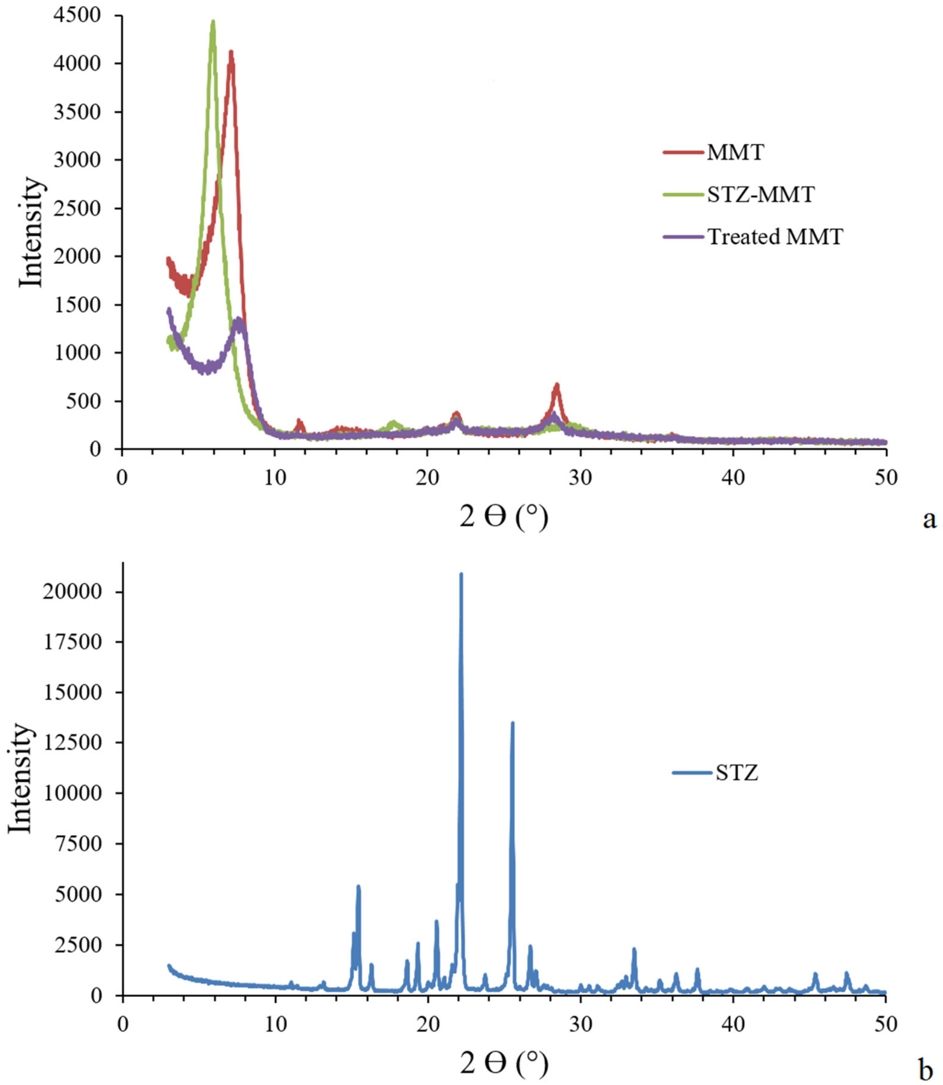

Figure S1. Powder X-ray diffraction patterns of the oriented aggregates of the MMT, STZ-MMT, Treated MMT (**a**) and STZ (**b**) samples.

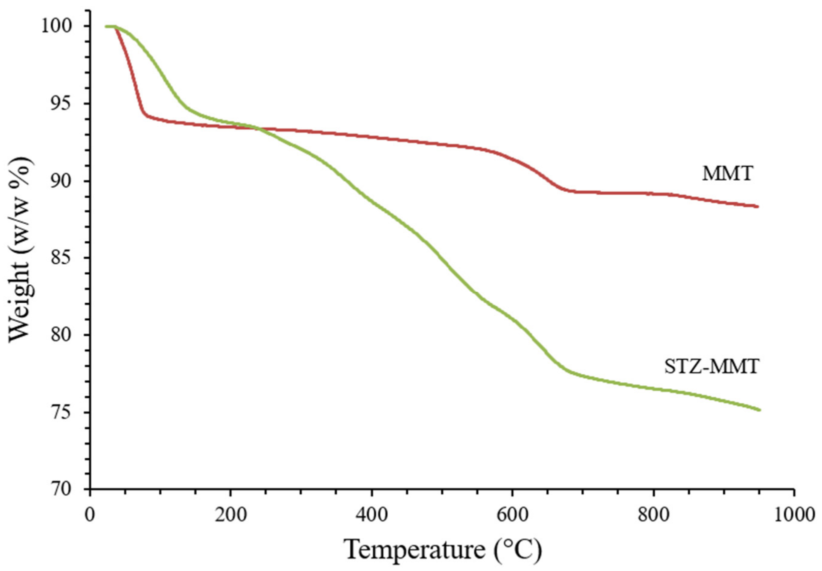

Figure S2. TGA profiles of MMT and STZ-MMT samples.

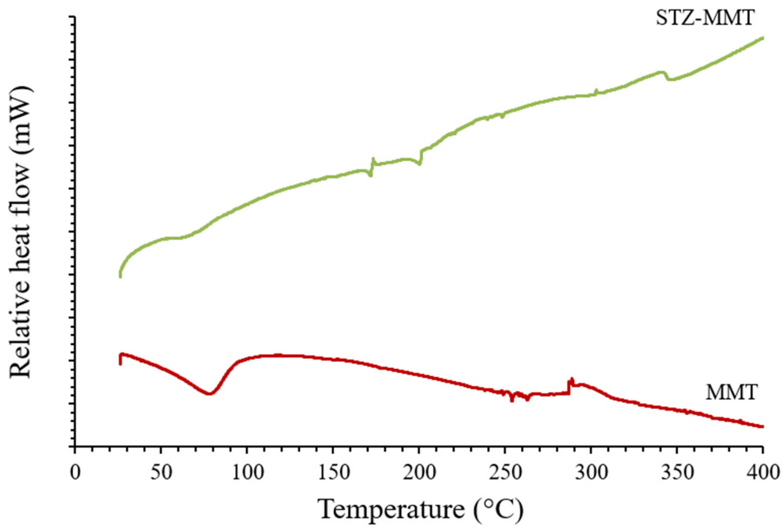

Figure S3. Enlarged DSC profiles of MMT and STZ-MMT.

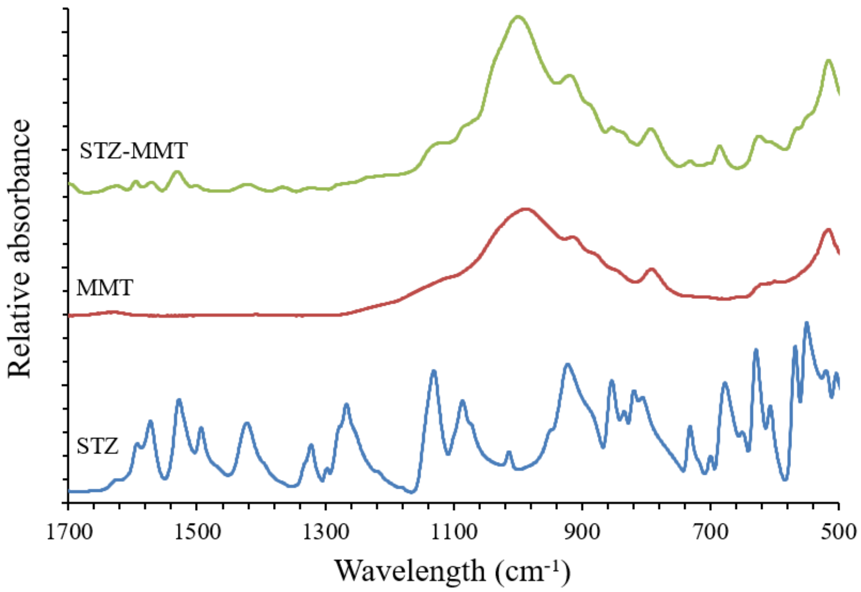

Figure S4. Zoom of the FTIR spectra of STZ, MMT and STZ-MMT.
